# Supplementary material for: The Distribution of Puumala orthohantavirus Genome Variants Correlates with the Regional Landscapes in the Trans-Kama Area of the Republic of Tatarstan
Source: Pathogens. 2021 Sep 10;10(9):1169. doi: 10.3390/pathogens10091169 (PMC8471081; doi:10.3390/pathogens10091169)
Supplement: Supplementary file 1 [file pathogens-10-01169-s001.zip › pathogens-1331703-supplementary.pdf]

Supplementary Data S1. List of *Puumala ortohantavirus* strains identified in the Trans-Kama area of the Republic of Tatarstan

PUUV/Aksarino/MG_108/2015
PUUV/Aksarino/MG_109/2015
PUUV/Almetievsk/MG_1182/2018
PUUV/Almetievsk/MG_1183/2018
PUUV/Almetievsk/MG_1185/2018
PUUV/Chistopolskie_Vyselki/MG_2030/2020
PUUV/Chistopolskie_Vyselki/MG_2031/2020
PUUV/Chistopolskie_Vyselki/MG_2032/2020
PUUV/Galaktionovo/MG_2037/2020
PUUV/Galaktionovo/MG_2038/2020
PUUV/Galaktionovo/MG_2039/2020
PUUV/Ilinka/MG_998/2017
PUUV/Jalil/MG_1998/2020
PUUV/Jalil/MG_2001/2020
PUUV/Kamyshly/MG_1196/2018
PUUV/Kamyshly/MG_1199/2018
PUUV/Kamyshly/MG_1200/2018
PUUV/Krasnyi Klyuch/MG_158/2015
PUUV/Kurkul/MG_1459/2019
PUUV/Kutemeli/MG_1992/2020
PUUV/Kyzyl-Yulskoe lesnichestvo/MG_1575/2019
PUUV/Naberezhnye_Chelny/MG_260/2015
PUUV/Nizhnekamsk/MG_134/2015
PUUV/Nizhnekamsk/MG_139/2015
PUUV/Nizhnekamsk/MG_1554/2019
PUUV/Nizhnekamsk/MG_1555/2019
PUUV/Nizhnekamsk/MG_415/2016
PUUV/Nizhnekamsk/MG_420/2016
PUUV/Nizhnekamsk/MG_436/2016
PUUV/Novyi/MG_1089/2017
PUUV/Oktiabrino/MG_1469/2019
PUUV/Poruchikovo/MG_074/2015
PUUV/Starye Salmany/MG_1586/2019
PUUV/Starye Salmany/MG_1589/2019
PUUV/Starye Salmany/MG_1590/2019
PUUV/Staryy Dryush/MG_1652/2019
PUUV/Tatarskoe Utiashkino/MG_1419/2019
PUUV/Verkhnee Almurzino/MG_1613/2019
PUUV/Zai-Karatai/MG_1206/2018
PUUV/Zai-Karatai/MG_1207/2018

Supplementary Data S2. List of *Puumala orthohantavirus* sequence references used for the phylogenetic analysis accessed from the GenBank database

Strain	Accession No
Segment S	
PUUV/Teteevo/MG_1041/2017	MZ441151
PUUV/Sotyi/MG_952/2017	MZ441152
PUUV/Mamadysh/MG_980/2017	MW504250
PUUV/Kazan/MG_845/2017	MW587804
PUUV/Lenino-Kokushkino/MG_1140/2017	MW504252
PUUV/Laishevo/MG_809/2017	MW504247
PUUV/Staraya_Pristan/MG_1055/2017	MZ441153
Samara_49/CG/2005	AB433843
Puu/Kazan	Z84204
CG1820	M32750
DTK/Ufa-97	AB297665
Sotkamo 2009	HE801633
PUUV/Pieksamaki/human_lung/2008	JN831947
Mu/07/1219	KJ994776
PUUV/Ardennes/Mg156/2011	KT247592
PUUV/Orleans/Mg29/2010	KT247595
Umea/hu	AY526219
Tula orthohantavirus	AF164093
Segment M	
PUUV/Teteevo/MG_1041/2017	MT495338
PUUV/Sotyi/MG_952/2017	MT495334
PUUV/Mamadysh/MG_980/2017	MT495336
PUUV/Kazan/MG_845/2017	MT495325
PUUV/Lenino-Kokushkino/MG_1140/2017	MT495344
PUUV/Laishevo/MG_809/2017	MT495353
PUUV/Staraya_Pristan/MG_1055/2017	MT495342
Samara_49/CG/2005	AB433850
Puu/Kazan	Z84205
CG1820	M29979
DTK/Ufa-97	AB297666
Sotkamo 2009	HE801634
PUUV/Pieksamaki/human_lung/2008	JN831948
Mu/07/1219	KJ994777
PUUV/Ardennes/Mg156/2011	KT247603
PUUV/Orleans/Mg29/2010	KT247601
Umea/hu	AY526218
Tula orthohantavirus	NC_005228
Segment L	
PUUV/Teteevo/MG_1041/2017	MT502404
PUUV/Sotyi/MG_952/2017	MT502400
PUUV/Mamadysh/MG_980/2017	MT502402
PUUV/Kazan/MG_845/2017	MT502391
PUUV/Lenino-Kokushkino/MG_1140/2017	MT502410
PUUV/Laishevo/MG_809/2017	MT502386
PUUV/Staraya_Pristan/MG_1055/2017	MT502408

Samara_49/CG/2005	AB574183
Puu/Kazan	EF405801
CG1820	KT885050
DTK/Ufa-97	AB297667
Sotkamo 2009	HE801635
PUUV/Pieksamaki/human_lung/2008	JN831949
Mu/07/1219	KJ994778
PUUV/Ardennes/Mg156/2011	KT247609
PUUV/Orleans/Mg29/2010	KT247605
Umea/hu	AY526217
Tula orthohantavirus	NC_005226

Supplementary Table S1. Grouping of bank vole trapping sites, samples/strains and clades/subclades

S segment				M segment				L segment			
Clade/ subclade	No group/ subgroup	Site	Sample/strain	Clade/ subclade	No group/ subgroup	Site	Sample/strain	Clade/ subclade	No group/ subgroup	Site	Sample/strain
A/A2	1	S1, S2, S3	260, 1089, 1652	A/A2	1	S1, S2, S3	260, 1089, 1652	A/A2	1	S1, S2, S3	260, 1089, 1652
	2/2a<br>2/2b	S4<br>S6, S7	158, 415, 420<br>139, 1554, 1575		2/2a<br>2/2b	S4<br>S6, S7	158, 415, 420<br>139, 1554, 1575		2/2a<br>2/2b	S4<br>S6, S7	158, 415, 420, 1555<br>139, 1554, 1575
	3 (N-K)	S5, S6	998, 134, 436, 1555		3 (N-K)	S5, S6	998, 134, 436, 1555	A/N-K	3 (N-K)	S5, S6	998, 134, 436
A/A1	4 (Z)	S8, S9	108, 109, 074	A/A1	4 (Z)	S8, S9	108, 109, 074	A/Z	4 (Z)	S8, S9	108, 109, 074
	5	S11	1998, 2001		5	S11	1998, 2001	A/A1	5	S11, S14	2001, 1199
A/A1	6	S12	1182, 1183, 1185	A/A1	6	S12	1182, 1183, 1185	A/A1	6	S12	1182, 1183, 1185
A/A1	7	S13, S14	1206, 1207, 1196, 1199, 1200	A/A1	7	S13, S14	1206, 1207, 1196, 1199, 1200	A/A1	7	S11, S13, S14	1998, 1206, 1207, 1196, 1200
A/A1	8	S10	1992	C	8	S10	1992	A	8	S10	1992
B/B1	10	S19, S20	1586, 1589, 1590, 1613	B/B1	10	S19, S20	1586, 1589, 1590, 1613	B/B1	10	S19, S20	1586, 1589, 1590, 1613
B/B2	9	S16, S17	2030, 2031, 2032, 2037, 2038, 2039	B/B2	9	S16, S17	2030, 2031, 2032, 2037, 2038, 2039	B/B2	9	S16, S17	2030, 2031, 2032, 2037, 2038, 2039
B/B2	11	S15	1419	B/B2	11	S15	1419	B/B2	11	S15	1419
B/B2	12	S21	1469	B/B2	13	S18	1459	B/B2	13	S18	1459
B/B2	13	S18	1459	B/B2	12	S21	1469	B/B2	12	S21	1469

Supplementary Table S2. Divergence of the PUUV S segment nucleotide sequences among the PUUV strain genetic groups in the Trans-Kama area (%).

Group	Site	Strain designation	1	2a	2b	3	4	5	6	7	8	9	10	11	12	13
1	S1, S2, S3	MG_260, MG_1089, MG_1652	0.0-2.0	2.2-2.8	2.4-2.9	1.4-2.0	4.1-4.7	4.4-4.8	4.4-5.0	4.4-5.0	4.3-4.9	4.1-4.7	3.9-4.4	4.6-5.0	4.1-4.6	4.8-5.3
2a	S4	MG_158, MG_415, MG_420		0.0-0.2	1.5-1.6	2.0-2.1	4.6	5.0	5.0	4.8-4.9	4.5	4.6-4.7	4.5-4.6	5.1	4.6	5.1
2b	S6, S7	MG_139, MG_1554, MG_1575			0.1-0.2	1.9-2.1	4.7-4.9	4.9-5.0	5.0-5.2	4.9-5.1	4.5-4.6	4.7-5.0	4.4-4.7	5.1-5.2	4.6	5.0-5.1
3	S5, S6	MG_998, MG_134, MG_436, MG_1555				0.0-0.1	3.7-4.0	4.2-4.3	4.4-4.5	4.1-4.4	4.0-4.1	3.9-4.1	3.6-4.0	4.6-4.7	3.9-4.0	4.5-4.6
4	S8, S9	MG_108, MG_109, MG_074					0.0-1.4	2.5-2.7	2.7-3.0	2.4-2.6	3.1-3.2	4.3-4.6	4.3-4.6	5.3-5.5	4.2-4.3	5.1-5.2
5	S11	MG_1998, MG_2001						0.1	1.1-1.3	0.8-0.9	3.2	4.6-4.7	4.6-4.8	5.8	4.6	5.1
6	S12	MG_1182, MG_1183, MG_1185							0.0-0.2	1.2-1.5	3.6-3.7	4.6-4.7	4.8-5.0	5.8-6.0	4.6	5.2
7	S13, S14	MG_1206, MG_1207, MG_1196, MG_1199, MG_1200								0.0-0.2	3.2-3.3	4.4-4.6	4.2-4.4	5.6-5.7	4.4-4.5	4.8-4.9
8	S10	MG_1992									-	5.0-5.1	4.5-4.8	5.5	4.6	5.4
9	S16, S17	MG_2030, MG_2031, MG_2032, MG_2037, MG_2038, MG_2039										0.0-0.2	2.8-3.2	2.1-2.3	1.3-1.5	2.3-2.4
10	S19, S20	MG_1586, MG_1589, MG_1590, MG_1613											0.0-1.4	3.8	2.8	3.2-3.4
11	S15	MG_1419												-	1.7	3.2
12	S21	MG_1469													-	2.0
13	S18	MG_1459														-

Supplementary Table S3. Nucleotide sequence divergence of the Trans-Kama PUUV strains compared to the Pre-Kama strains and strains of the RUS, FIN, CE, and N-SCA genetic lineages.

Group	Segment	RUS											FIN		CE			N-SCA
		Pre-Kama																
		PUUV/Teteevo/MG_1041/2017	PUUV/Sotyi/MG_952/2017	PUUV/Mamadysh/MG_980/2017	PUUV/Kazan/MG_845/2017/	PUUV/Lenino-Kokushkino/MG_1140/2017	PUUV/Laishevo/MG_809/2017	PUUV/Staraya_Pristan/MG_1055/2017	Samara_49/CG/2005	Puu/Kazan	CG1820	DTK/Ufa-97	Sotkamo_2009	PUUV/Pieksamaki/human_kidney/2008	Mu/07/1219	PUUV/Ardennes/Mg75/2011	PUUV/Orleans/Mg29/2010	strain_Umea/hu
1	S	4.8-5.1	5.5-6.1	5.1-5.7	5.8-6.2	5.9-6.2	5.1-5.6	5.0-5.5	4.2-4.9	4.7-5.3	6.2-6.8	6.1-6.7	17.2-17.7	16.6-17.2	17.9-18.3	19.5-19.7	18.9-19.5	18.2-18.4
	M	5.6	4.3-4.6	5.1	6.1-6.3	6.0-6.2	6.4-6.5	6.4-6.7	6.7-7.0	6.0-6.2	15.7-16.1	14.9-15.3	17.6-17.7	18.8-19.3	25.3-25.4	21.8-22.1	25.2-26.1	22.8-23.1
	L	5.6-6.8	6.3-7.3	5.8-6.8	6.2-6.8	6.2-6.8	7.3-8.0	7.6-7.7	4.8-6.0	7.3-7.8	15.3-16.7	15.3-16.7	20.1-20.6	19.9-20.3	22.8-23.7	22.9-24.0	21.1-22.2	24.8-25.8
2a	S	5.2	6.2	5.7	6.3	6.4	5.7	5.6	5.0	5.6	6.2	6.1	16.8-17.0	16.0-16.2	18.8-19.0	19.1-19.3	19.4	19.2-19.4
	M	5.7-5.8	4.8-4.9	5.2-5.3	6.4-6.5	6.2-6.3	7.1-7.2	7.3-7.4	7.0-7.1	6.5-6.6	16.0-16.1	15.1-15.3	16.6-16.7	18.4-18.6	24.8-24.9	21.7-21.8	25.2-25.4	22.5-22.7
	L	6.0-6.1	6.6-6.8	6.1-6.3	6.5-7.0	6.5-7.0	8.0-8.5	8.0-8.5	5.5-6.0	7.1-7.3	15.0	15.0	20.7-20.9	20.1-20.3	23.7-24.1	23.2	21.8-22.0	25.8
2b	S	5.4-5.5	6.5-6.6	5.6-5.8	6.1-6.2	6.3-6.4	6.0-6.1	5.9-6.0	5.0-5.1	5.7-5.8	6.2-5.3	6.1-6.2	16.4-16.5	16.0-16.1	18.0-18.1	19.1-19.2	19.2-19.3	18.3-18.5
	M	5.8-5.9	4.9-5.0	5.0-5.1	6.6-6.7	6.5-6.6	6.8-7.0	7.1-7.2	7.0-7.1	6.4-6.6	16.0-16.1	15.1-15.3	16.4-16.6	18.0-18.2	25.4-25.6	21.8-22.0	25.7-25.8	22.7-22.9
	L	6.1	6.8	6.6	7.0	7.0	8.5	8.5	6.0	7.5-7.6	15.2	15.2	21.2	20.3	23.2	23.4	21.6	25.1
3	S	4.7-4.8	5.6-5.7	5.1-5.2	5.7-5.8	5.8-5.9	5.3-5.4	5.2-5.3	4.2-4.3	5.1	5.5	5.4-5.5	16.0-17.0	16.2-16.3	18.0-18.1	19.3-19.4	18.9-19.0	18.3-18.4
	M	5.8-6.1	4.6-4.9	5.0-5.3	6.5-6.8	6.6-6.9	6.9-7.3	7.2-7.5	7.1-7.4	6.0-6.3	16.3-16.4	15.6-15.7	18.2-18.6	19.1-19.5	24.9-25.0	22.4-22.8	25.5-26.0	23.5-23.7
	L	6.1-6.3	6.5-6.6	6.1-6.3	6.6-6.8	6.6-6.8	8.5-8.6	8.5-8.7	6.3	6.8-7.0	15.0-15.1	15.0-15.1	20.1-20.3	20.3-20.5	24.0-24.2	23.4-23.6	21.6-21.8	25.1-25.4
4	S	4.9-5.1	5.6-6.0	5.0-5.1	5.4-5.8	5.7-6.1	5.5-5.6	5.4-5.5	4.6-4.7	5.6-5.8	6.3	6.2	17.0	16.5-16.8	17.8	18.8	19.5-20.1	18.4-18.6
	M	5.8-6.0	5.0-5.4	4.9-5.2	6.2-6.7	6.1-6.6	6.3-7.1	6.5-7.3	6.9-7.2	6.2-6.4	16.1-16.4	15.3-15.5	17.0-17.4	18.7	25.0-25.2	23.0-23.3	24.8-25.0	23.2-23.5
	L	6.5-7.1	7.0-7.1	6.5-7.0	7.5-8.0	7.5-8.0	8.7-9.2	9.0-9.5	6.1-6.8	7.5-8.1	15.5-15.7	15.5-15.7	19.6-20.9	20.7-20.8	23.2-23.3	23.9-24.2	21.6-22.6	25.0-25.3
5	S	5.1	5.7	5.5	6.3	6.6	6.1	6.0	4.8	5.8	6.6	6.5-6.6	17.0	16.2	17.9	18.6	19.9-20.0	18.4-18.5
	M	5.1	4.7	5.0	6.5	6.4	6.6	6.8	7.0	5.8	16.2	15.3	17.3	19.4	24.6	22.8	25.5	23.6
	L	5.8	5.8	5.8	6.0	6.0	7.5	7.5	5.3	6.8	14.9	14.9	19.6	20.1	23.8	23.6	22.0	24.8
6	S	5.0	6.0	5.4	6.4	6.7	6.0	5.9	5.0	5.9-6.1	6.9	6.8	16.9-17.0	16.2	17.4-17.6	18.0	19.6-19.7	18.2-18.4
	M	5.8	5.1	5.2	6.7	6.6	6.8	7.0	7.2	6.4	16.4	15.6	17.0	18.6	24.2	22.7	25.0	23.4
	L	5.5	5.8	5.8	6.0	6.0	7.5	7.5	5.9	6.4	15.5	15.5	19.8	20.4	24.0	23.1	21.6	25.5
7	S	5.0-5.1	5.7-5.8	5.3-5.4	6.3-6.4	6.6-6.7	6.1	6.0-6.1	4.6-4.7	5.8-5.9	6.5-6.7	6.5-6.6	17.1-17.2	16.2-16.3	18.0	18.2	20.0	18.4-18.5

	M	5.3	4.9	5.2	6.7	6.6	6.6	6.8	7.2	5.8	15.9	15.1	17.0	19.4	24.5	22.8	26.0	23.3
	L	6.5	6.5	6.5	6.3	6.3	7.8	7.8	6.0	7.1	14.9	14.9	20.1	20.2	24.0	23.3	21.7	25.8
8	S	5.3	5.8	5.3	6.5	6.7	6.1	6.0	5.2	5.9	6.4	6.3	16.0	16.4	16.8	18.7	19.4	17.9
	M	5.9	4.8	4.7	7.2	7.1	7.2	7.2	7.4	7.1	16.2	15.4	19.1	19.6	25.6	21.6	24.4	23.8
	L	6.0	7.6	7.3	6.5	6.5	7.6	7.6	6.4	8.1	14.7	14.7	20.0	20.2	23.5	23.1	22.6	26.7
9	S	2.6-2.8	5.1-5.2	4.8-4.9	5.4-5.5	5.5-5.6	4.6-4.7	4.6	3.7-3.9	4.9-5.0	5.7-5.8	5.6-5.7	17.1-17.3	15.3-15.5	18.3-18.5	18.9-19.1	19.6-19.9	17.2-17.4
	M	3.9-4.3	6.9-7.1	7.1-7.3	8.4-8.6	8.3-8.5	8.3-8.5	8.5-8.7	9.1-9.3	7.2-7.4	17.7-18.0	16.9-17.2	19.4-19.7	19.5-19.8	24.6-25.0	23.5-23.8	25.0-25.3	23.6-23.9
	L	3.4	7.5-7.8	7.5-7.8	7.2-7.3	7.2-7.3	8.7-8.9	8.5-8.7	7.6-7.8	7.8-8.2	15.2-15.4	15.2-15.4	20.1-20.5	19.3-19.7	24.3-24.5	21.6-22.1	21.4-21.6	24.7-24.9
10	S	3.4-3.9	5.0-5.4	4.6-4.9	4.9-5.1	5.0-5.1	4.5-4.6	4.4-4.6	3.6-3.8	4.6-5.0	5.8-6.0	5.7-5.9	16.5-16.7	15.5-15.6	18.9-19.4	18.5-19.3	19.7-19.8	17.7-17.9
	M	5.9-6.2	6.1-6.2	6.2-6.3	7.7-7.8	7.6-7.7	7.7-7.8	7.9-8.1	8.1-8.5	7.3-7.5	17.2-17.4	16.4-16.5	17.4-18.1	18.8-18.9	24.6	24.1-24.2	24.6-25.1	24.1-24.5
	L	4.2	7.3-7.7	7.5-7.6	6.5-6.8	6.5-6.8	8.3	8.2	6.6	5.8-6.0	15.5-15.6	15.5-15.6	20.4-20.6	20.0-20.5	23.6-24.0	12.6-21.8	21.3	26.0-26.1
11	S	3.6	6.0	5.5	6.2	6.3	5.5	5.4	4.6	5.4	6.0	5.9	17.2	15.9	18.1	18.4	19.9	17.4
	M	3.7	5.9	5.9	7.8	7.7	7.2	7.4	8.2	7.6	16.5	15.7	18.3	19.1	24.6	23.2	24.2	24.1
	L	3.4	7.6	7.1	7.5	7.5	8.7	8.5	8.6	8.6	16.3	16.3	20.9	20.7	23.8	22.5	21.8	25.0
12	S	2.8	5.4	4.9	5.5	5.6	4.9	4.8	3.7	5.1	5.6	5.6	17.0	15.3	17.7	18.4	19.4	17.1
	M	3.2	5.7	6.1	7.4	7.3	7.1	7.3	8.1	6.2	17.2	16.2	18.8	18.8	24.9	22.6	24.1	23.3
	L	3.2	7.2	7.2	6.7	6.7	8.2	8.0	6.9	7.3	15.7	15.7	20.2	19.3	23.0	22.7	21.8	25.2
13	S	3.3	5.8	5.3	5.5	5.7	5.4	5.3	4.0	5.6	6.0	5.9	16.4	15.5	19.0	18.7	19.6	17.8
	M	4.0	6.9	6.8	7.6	7.5	7.8	8.0	8.7	7.2	17.5	16.6	18.2	18.3	24.9	21.9	24.2	23.5
	L	2.5	6.7	6.7	6.5	6.5	8.0	7.9	7.0	6.8	15.8	15.8	20.3	19.7	24.1	22.5	21.4	24.9

Supplementary Table S4. Divergence of the PUUV M segment nucleotide sequences among the PUUV strain genetic groups in the Trans-Kama area (%).

Group	Site	Strain designation	1	2a	2b	3	4	5	6	7	8	9	10	11	12	13
1	S1, S2, S3	MG_260, MG_1089, MG_1652	0.7-1.9	1.8-2.6	2.1-2.5	1.9-2.9	4.4-4.8	3.5-3.8	4.0-4.4	3.8-4.0	4.5-4.9	5.9-7.0	5.3-6.2	5.7-6.1	5.2-6.0	6.1-6.7
2a	S4	MG_158, MG_415, MG_420		0.0-0.1	1.1-1.3	2.3-2.7	4.6-5.0	3.8-3.9	4.4-4.5	4.2-4.3	4.5-4.6	6.2-6.5	5.5-6.0	5.7-5.8	5.3-5.4	6.1-6.2
2b	S6, S7	MG_139, MG_1554, MG_1575			0.1-0.2	2.4-2.8	4.3-4.9	3.9-4.0	4.3-4.4	4.1-4.2	4.6-4.7	6.1-6.4	5.7-6.2	5.5-5.7	5.3-5.4	6.0-6.1
3	S5, S6	MG_998, MG_134, MG_436, MG_1555				0.0-0.3	4.2-4.8	3.6-4.0	4.1-4.4	4.1-4.4	4.4-4.7	5.9-6.4	5.3-6.4	5.5-5.9	5.3-5.6	6.1-6.4
4	S8, S9	MG_108, MG_109, MG_074					0.0-1.3	2.6-2.7	2.6-2.7	3.0-3.1	4.7-5.1	6.0-6.2	5.5-5.9	5.7-5.8	5.2	5.9-6.0
5	S11	MG_1998, MG_2001						0.0	1.0	0.8	4.4	5.4-5.7	5.1-5.2	4.8	4.7	5.3
6	S12	MG_1182, MG_1183, MG_1185							0.0	1.4	4.8	6.1-6.3	5.4-5.5	5.0	5.3	5.9
7	S13, S14	MG_1206, MG_1207, MG_1196, MG_1199, MG_1200								0.0	4.8	5.7-5.9	5.4-5.6	5.0	4.7	5.5
8	S10	MG_1992									-	6.6-6.9	5.6-5.9	5.7	5.7	6.1
9	S16, S17	MG_2030, MG_2031, MG_2032, MG_2037, MG_2038, MG_2039										0.0-0.4	6.5-6.8	3.8-4.2	2.2-2.4	4.0-4.2
10	S19, S20	MG_1586, MG_1589, MG_1590, MG_1613											0.0-2.6	5.3-6.1	6.1-6.2	6.2-6.7
11	S15	MG_1419												-	2.7	3.7
12	S21	MG_1469													-	3.0
13	S18	MG_1459														-

Supplementary Table S5. Divergence of the PUUV L segment nucleotide sequences among the PUUV strain genetic groups in the Trans-Kama area (%).

Group	Site	Strain designation	1	2a	2b	3	4	5	6	7	8	9	10	11	12	13
1	S1, S2, S3	MG_260, MG_1089, MG_1652	0.6-2.0	1.8-3.2	2.3-2.9	3.1-4.4	3.5-5.0	3.2-4.2	3.2-4.0	3.6-4.2	4.3-5.1	6.3-7.1	5.3-6.1	7.0-7.8	5.8-6.6	5.7-6.6
2a	S4	MG_158, MG_415, MG_420, **MG_1555**		0.0-1.1	0.8-1.5	3.4-4.4	3.9-4.7	3.2-4.0	3.2-4.0	3.2-4.0	4.7-5.1	6.0-6.5	5.8-6.1	7.0-7.1	5.8-6.0	5.7-6.2
2b	S6, S7	MG_139, MG_1554, MG_1575			0.0	3.9-4.0	4.7	3.7	3.7	3.7	5.2	6.5-6.7	6.0-6.1	7.1	6.3	6.2
3	S5, S6	MG_998, MG_134, MG_436				0.0-0.2	4.4-4.5	2.8-2.9	2.8-2.9	2.8-2.9	5.1-5.3	6.8-7.0	6.1-6.8	7.8-8.0	6.3-6,5	5.8-6.0
4	S8, S9	MG_108, MG_109, MG_074					0.0-3.1	4.5-4.8	4.3-4.7	4.8	5.8-6.1	7.3-8.3	7.0-7.8	8.1-9.1	6.6-7.6	6.8-7.5
5	S11	MG_2001, **MG_1199**						0.0	1.4	1.2	4.5	6.0-6.1	5.7-6.1	7.8	5.8	5.5
6	S12	MG_1182, MG_1183, MG_1185							0.0	1.5	4.2	5.8-6.0	5.8-6.3	7.1	5.3	5.2
7	S13, S14	**MG_1998**, MG_1206, MG_1207, MG_1196, MG_1200								0.0	4.5	6.5-6.6	6.0-6.1	7.8	6.3	5.8
8	S10	MG_1992									-	6.8	6.1-6.3	7.6	6.1	6.3
9	S16, S17	MG_2030, MG_2031, MG_2032, MG_2037, MG_2038, MG_2039										0.0-0.3	4.5-4.8	4.2-4.3	2.3-2.6	2.3-2.5
10	S19, S20	MG_1586, MG_1589, MG_1590, MG_1613											0.0-1.5	5.1-5.6	4.2-4.4	4.0-4.2
11	S15	MG_1419												-	4.2	3.4
12	S21	MG_1469													-	2.3
13	S18	MG_1459														-

[a] The designation of strains whose segment L sequence is included in other group compared to the segment S and M sequences are highlighted in bold

Supplementary Table S6. Divergence of the PUUV N protein, partial glycoprotein precursor and partial RdRp aa sequences among the different groups (%)

Group	aa sequence	1	2a	2b	3	4	5	6	7	8	9	10	11	12	13
1	N protein	0.2-0.5	0.0-0.5	0.0-0.5	0.0-0.5	0.0-0.7	0.0-0.5	0.0-0.5	0.0-0.7	0.2-0.7	0.0-0.5	0.0-0.5	0.2-0.7	0.0-0.5	0.0-0.5
	Gn-Gc precursor	0.0-0.6	0.3-0.9	0.6-1.2	0.3-1.2	0.3-0.9	0.0-0.6	0.0-0.6	0.0-0.6	1.2-1.8	0.6-1.5	0.0-0.6	0.3-0.9	0.3-0.9	0.0-0.6
	RdRp	0.0-0.5	1.4-2.3	1.8-2.3	1.4-2.3	1.4-2.3	1.4-1.8	0.9-1.4	0.9-1.4	0.9-1.4	1.4-2.3	1.4-2.3	0.9-1.4	0.9-1.4	1.8-2.3
2a	N protein		0.0	0.0	0.0	0.0-0.2	0.0	0.0	0.0-0.2	0.2	0.0	0.0	0.2	0.0	0.0
	Gn-Gc precursor		0.0	0.3-0.6	0.6-0.9	0.6	0.3	0.3	0.3	1.5	0.9-1.2	0.3-0.6	0.6	0.6	0.3
	RdRp		0.0-0.5	0.5-0.9	0.9-1.8	0.9-1.8	0.9-1.4	0.5-0.9	0.5-0.9	0.5-0.9	0.9-1.8	0.9-1.8	0.5-0.9	0.5-0.9	1.4-1.8
2b	N protein			0.0	0.0	0.0-0.2	0.0	0.0	0.0-0.2	0.2	0.0	0.0	0.2	0.0	0.0
	Gn-Gc precursor			0.0-0.3	0.9-1.5	0.9-1.2	0.6-0.9	0.6-0.9	0.6-0.9	1.8-2.1	1.2-1.8	0.6-1.2	0.9-1.2	0.9-1.2	0.6-0.9
	RdRp			0.0	1.4-1.8	1.4-1.8	1.4	0.9	0.9	0.9	1.4-1.8	1.4-1.8	0.9	0.9	1.8
3	N protein				0.0	0.0-0.2	0.0	0.0	0.0-0.2	0.2	0.0	0.0	0.2	0.0	0.0
	Gn-Gc precursor				0.0-0.3	0.6-0.9	0.3-0.6	0.3-0.6	0.3-0.6	1.5-1.8	0.9-1.5	0.3-0.9	0.6-0.9	0.6-0.9	0.3-0.6
	RdRp				0.0-0.5	0.9-1.8	0.9-1.4	0.5-0.9	0.5-0.9	0.5-0.9	0.9-1.8	0.9-1.8	0.5-0.9	0.5-0.9	1.4-1.8
4	N protein					0.0-0.2	0.0-0.2	0.0-0.2	0.0-0.5	0.2-0.5	0.0-0.2	0.0-0.2	0.2-0.5	0.0-0.2	0.0-0.2
	Gn-Gc precursor					0.0-0.6	0.3	0.3	0.3	1.5	0.9-1.2	0.3-0.6	0.6	0.6	0.3
	RdRp					0.0-0.5	0.9-1.4	0.5-0.9	0.5-0.9	0.5-0.9	0.9-1.8	0.9-1.8	0.5-0.9	0.5-0.9	1.4-1.8
5	N protein						0.0	0.0	0.0-0.2	0.2	0.0	0.0	0.2	0.0	0.0
	Gn-Gc precursor						0.0	0.0	0.0	1.2	0.6-0.9	0.0-0.3	0.3	0.3	0.0
	RdRp						0.0	0.5	0.5	0.5	0.9-1.4	0.9-1.4	0.5	0.5	1.4
6	N protein							0.0	0.0-0.2	0.2	0.0	0.0	0.2	0.0	0.0
	Gn-Gc precursor							0.0	0.0	1.2	0.6-0.9	0.0-0.3	0.3	0.3	0.0
	RdRp							0.0	0.0	0.0	0.5-0.9	0.5-0.9	0.0	0.0	0.9
7	N protein								0.0-0.2	0.2-0.5	0.0-0.2	0.0-0.2	0.2-0.5	0.0-0.2	0.0-0.2

	Gn-Gc precursor<br>RdRp								0.0<br>0.0	1.2<br>0.0	0.6-0.9<br>0.5-0.9	0.0-0.3<br>0.5-0.9	0.3<br>0.0	0.3<br>0.0	0.0<br>0.9
8	N protein<br>Gn-Gc precursor<br>RdRp									-<br>-<br>-	0.2<br>1.8-2.1<br>0.5-0.9	0.2<br>1.2-1.5<br>0.5-0.9	0.0<br>1.5<br>0.0	0.2<br>1.5<br>0.0	0.2<br>1.2<br>0.9
9	N protein<br>Gn-Gc precursor<br>RdRp										0.0<br>0.0-0.6<br>0.0-0.5	0.0<br>0.6-1.2<br>0.9-1.8	0.2<br>0.9-1.2<br>0.5-0.9	0.0<br>0.3-0.6<br>0.5-0.9	0.0<br>0.6-0.9<br>1.4-1.8
10	N protein<br>Gn-Gc precursor<br>RdRp											0.0<br>0.0-0.3<br>0.0-0.5	0.2<br>0.3-0.6<br>0.5-0.9	0.0<br>0.3-0.6<br>0.5-0.9	0.0<br>0.0-0.3<br>1.4-1.8
11	N protein<br>Gn-Gc precursor<br>RdRp												-<br>-<br>-	0.2<br>0.6<br>0.0	0.2<br>0.3<br>0.9
12	N protein<br>Gn-Gc precursor<br>RdRp													-<br>-<br>-	0.0<br>0.3<br>0.9
13	N protein<br>Gn-Gc precursor<br>RdRp														-<br>-<br>-

Supplementary Table S7. Divergence of the PUUV N protein, partial glycoprotein precursor and partial RdRp aa sequences of the Trans-Kama area strains to the RUS, FIN, CE and N-SCA genetic lineage strains

Group	aa sequence	RUS											FIN		CE			S-SCA
		Pre-Kama area																
		PUUV/Teteevo/MG_1041/2017	PUUV/Sotyi/MG_952/2017	PUUV/Mamadysh/MG_980/2017	PUUV/Kazan/MG_845/2017	PUUV/Lenino-Kokushkino/MG_1140/2017	PUUV/Laishevo/MG_809/2017	PUUV/Staraya_Pristan/MG_1055/2017	Samara_49/CG/2005	Puu/Kazan	CG1820	DTK/Ufa-97	Sotkamo_2009	PUUV/Pieksamaki/human_kidney/2008	Mu/07/1219	PUUV/Ardennes/Mg75/2011	PUUV/Orleans/Mg29/2010	Umea/hu
1	N protein	0.0-0.5	0.5-0.9	0.5-0.9	0.5-0.9	0.5-0.9	0.5-0.9	0.5-0.9	0.0-0.5	0.7-0.9	0.9-1.4	0.7-1.2	3.3-3.8	28-3.3	2.6-3.1	3.6-4.0	4.0-4.5	3.8-4.3
	Gn-Gc precursor	0.6-1.2	0.0-0.6	0.3-0.9	0.6-1.2	0.3-0.9	0.3-0.9	0.3-0.9	0.6-1.2	0.3-0.9	3.0-3.6	1.5-2.1	3.0-3.6	3.0-3.6	8.6-9.3	7.0-7.7	7.7-8.3	8.3-9.0
	RdRp	1.4-1.8	0.9-1.4	0.9-1.4	0.9-1.4	0.9-1.4	0.9-1.4	1.4-1.8	1.8-2.3	1.4-1.8	2.3-2.8	2.3-2.8	6.5-7.0	7.0-7.5	9.5-10.0	8.0-8.5	7.5-8.0	10.5-11.0
2a	N protein	0.0	0.5	0.5	0.5	0.5	0.5	0.5	0.0	0.9	0.9	0.7	3.3	2.8	2.6	3.6	4.0	3.8
	Gn-Gc precursor	0.9	0.3	0.6	0.9	0.6	0.6	0.6	0.9	0.6	2.7	1.2	2.7	2.7	8.3	6.7	7.4	8.0
	RdRp	0.9-1.4	0.5-0.9	0.5-0.9	0.5-0.9	0.5-0.9	0.5-0.9	0.9-1.4	1.4-1.8	0.9-1.4	1.8-2.3	1.8-2.3	6.5-7.0	7.0-7.5	9.5-10.0	8.0-8.5	7.5-8.0	11.0-11.5
2b	N protein	0.0	0.5	0.5	0.5	0.5	0.5	0.5	0.0	0.9	0.9	0.7	3.3	2.8	2.6	3.6	4.0	3.8
	Gn-Gc precursor	1.2-1.5	0.6-0.9	0.9-1.2	1.2-1.5	0.9-1.2	0.9-1.2	0.9-1.2	1.2-1.5	0.9-1.2	3.0-3.3	1.5-1.8	3.0-3.3	3.0-3.3	8.6-9.0	7.0-7.4	7.7-8.0	8.3-8.6
	RdRp	1.4	0.9	0.9	0.9	0.9	0.9	1.4	1.8	1.4	2.3	2.3	7.0	7.5	10.0	8.5	8.0	11.5

	N protein	0.0	0.5	0.5	0.5	0.5	0.5	0.5	0.0	0.9	0.9	0.7	3.3	2.8	2.6	3.6	4.0	3.8
3	Gn-Gc precursor	0.9-1.2	0.3-0.6	0.6-0.9	0.9-1.2	0.6-0.9	0.6-0.9	0.6-0.9	0.9-1.2	0.6-0.9	3.0-3.3	1.5-1.8	3.0-3.3	3.0-3.3	8.6-9.0	7.0-7.4	7.7-8.0	8.3-8.6
	RdRp	0.9-1.4	0.5-0.9	0.5-0.9	0.5-0.9	0.5-0.9	0.5-0.9	0.9-1.4	1.4-1.8	0.9-1.4	1.8-2.3	1.8-2.3	6.5-7.0	7.0-7.5	9.5-10.0	8.0-8.5	7.5-8.0	10.0-10.5
	N protein	0.0-0.2	0.5-0.7	0.5-0.7	0.5-0.7	0.5-0.7	0.5-0.7	0.5-0.7	0.0-0.2	0.9-1.2	0.9-1.2	0.7-0.9	3.3-3.6	2.8-3.1	2.6-2.8	3.8	4.0-4.3	3.8-4.0
4	Gn-Gc precursor	0.9	0.3	0.6	0.9	0.6	0.6	0.6	0.9	0.6	3.0-3.3	1.5-1.8	3.3	3.3	9.0	7.4	7.7-8.0	8.3-8.6
	RdRp	0.9-1.4	0.5-0.9	0.5-0.9	0.5-0.9	0.5-0.9	0.5-0.9	0.9-1.4	1.4-1.8	0.9-1.4	1.8-2.3	1.8-2.3	6.1-6.5	7.0-7.5	8.5-9.0	8.0-8.5	7.5-8.0	11.0-11.5
	N protein	0.0	0.5	0.5	0.5	0.5	0.5	0.5	0.0	0.9	0.9	0.7	3.3	2.8	2.6	3.6	4.0	3.8
5	Gn-Gc precursor	0.6	0.0	0.3	0.6	0.3	0.3	0.3	0.6	0.3	3.0	1.5	3.0	3.0	8.6	7.0	7.7	8.3
	RdRp	0.9	0.5	0.5	0.5	0.5	0.5	0.9	1.4	0.9	1.8	1.8	6.5	7.0	9.0	8.0	7.5	10.5
	N protein	0.0	0.5	0.5	0.5	0.5	0.5	0.5	0.0	0.9	0.9	0.7	3.3	2.8	2.6	3.6	4.0	3.8
6	Gn-Gc precursor	0.6	0.0	0.3	0.6	0.3	0.3	0.3	0.6	0.3	3.0	1.5	3.0	3.0	8.6	7.0	7.7	8.3
	RdRp	0.5	0.0	0.0	0.0	0.0	0.0	0.5	0.9	0.5	1.4	1.4	6.1	6.5	9.0	7.5	7.0	10.5
	N protein	0.0-0.2	0.5-0.7	0.5-0.7	0.5-0.7	0.5-0.7	0.5-0.7	0.5-0.7	0.0-0.2	0.9-1.2	0.9-1.2	0.7-0.9	3.3-3.6	2.8-3.1	2.6-2.8	3.6-3.8	4.0-4.3	3.8-4.0
7	Gn-Gc precursor	0.6	0.0	0.3	0.6	0.3	0.3	0.3	0.6	0.3	3.0	1.5	3.0	3.0	8.6	7.0	7.7	8.3
	RdRp	0.5	0.0	0.0	0.0	0.0	0.0	0.5	0.9	0.5	1.4	1.4	6.1	6.5	9.0	7.5	7.0	10.5
	N protein	0.2	0.2	0.2	0.7	0.7	0.2	0.2	0.2	0.7	0.7	0.5	3.1	2.6	2.4	3.3	4.0	3.6
8	Gn-Gc precursor	1.8	1.2	1.5	1.8	1.5	1.5	1.5	1.8	1.5	3.9	2.4	3.6	3.6	9.6	8.0	8.6	9.3
	RdRp	0.5	0.0	0.0	0.0	0.0	0.0	0.5	0.9	0.5	1.4	1.4	6.1	6.5	9.0	7.5	7.0	10.5
	N protein	0.0	0.5	0.5	0.5	0.5	0.5	0.5	0.0	0.9	0.9	0.7	3.3	2.8	2.6	3.6	4.0	3.8
9	Gn-Gc precursor	1.2-1.5	0.6-0.9	0.9-1.2	0.6-0.9	0.3-0.6	0.3-0.6	0.3-0.6	1.2-1.5	0.9-1.2	3.6-3.9	2.1-2.4	3.6-3.9	3.6-3.9	9.0-9.3	7.4-7.7	8.0-8.3	9.0-9.3
	RdRp	0.9-1.4	0.5-0.9	0.5-0.9	0.5-0.9	0.5-0.9	0.5-0.9	0.9-1.4	1.4-1.8	0.9-1.4	1.8-2.3	1.8-2.3	6.1-6.5	6.5-7.0	9.0-9.5	7.5-8.0	7.0-7.5	10.5
	N protein	0.0	0.5	0.5	0.5	0.5	0.5	0.5	0.0	0.9	0.9	0.7	3.3	2.8	2.6	3.6	4.0	3.8
10	Gn-Gc precursor	0.6-0.9	0.0-0.3	0.3-0.6	0.6-0.9	0.3-0.6	0.3-0.6	0.3-0.6	0.6-0.9	0.3-0.6	3.0-3.3	1.5-1.8	3.0	3.0	8.6-9.0	7.0-7.4	7.7-8.0	8.3
	RdRp	0.9-1.4	0.5-0.9	0.5-0.9	0.5-0.9	0.5-0.9	0.5-0.9	0.9-1.4	1.4-1.8	0.9-1.4	1.8-2.3	1.8-2.3	5.6-6.1	6.1-6.5	8.5-9.0	7.0-7.5	6.5-7.0	10.0-10.5
	N protein	0.2	0.2	0.2	0.7	0.7	0.2	0.2	0.2	0.7	0.7	0.5	3.1	2.6	2.4	3.3	3.8	3.6
11	Gn-Gc precursor	0.9	0.3	0.6	0.9	0.6	0.6	0.6	0.9	0.6	3.3	1.8	3.3	3.3	9.0	7.4	8.0	8.6
	RdRp	0.5	0.0	0.0	0.0	0.0	0.0	0.5	0.9	0.5	1.4	1.4	6.1	6.5	9.0	7.5	7.0	10.5

12	N protein	0.0	0.5	0.5	0.5	0.5	0.5	0.5	0.0	0.9	0.9	0.7	3.3	2.8	2.6	3.6	4.0	3.8
	Gn-Gc precursor	0.9	0.3	0.6	0.9	0.6	0.6	0.6	0.9	0.6	3.3	1.8	3.3	3.3	9.0	7.4	8.0	8.6
	RdRp	0.5	0.0	0.0	0.0	0.0	0.0	0.5	0.9	0.5	1.4	1.4	6.1	6.5	9.0	7.5	7.0	11.5
13	N protein	0.0	0.5	0.5	0.5	0.5	0.5	0.5	0.0	0.9	0.9	0.7	3.3	2.8	2.6	3.6	4.0	3.8
	Gn-Gc precursor	0.6	0.0	0.3	0.6	0.3	0.3	0.3	0.6	0.3	3.0	1.5	3.0	3.0	8.6	7.0	7.7	8.3
	RdRp	1.4	0.9	0.9	0.9	0.9	0.9	1.4	1.8	1.4	2.3	2.3	7.0	7.5	10.0	8.5	8.0	10.5
